# Development of Antimicrobial Laser-Induced Photodynamic Therapy Based on Ethylcellulose/Chitosan Nanocomposite with 5,10,15,20-Tetrakis(*m*-Hydroxyphenyl)porphyrin

**DOI:** 10.3390/molecules26123551

**Published:** 2021-06-10

**Authors:** Mohamed S. Hasanin, Mohamed Abdelraof, Mohamed Fikry, Yasser M. Shaker, Ayman M. K. Sweed, Mathias O. Senge

**Affiliations:** 1 Cellulose & Paper Department, National Research Centre, 33 El Bohouth St. (Former El Tahrir St.), Giza P.O. 12622, Egypt; sido_sci@yahoo.com; 2 Microbial Chemistry Department, Genetic Engineering and Biotechnology Research Division, National Research Centre, 33 El Bohouth St. (Former El Tahrir St.), Giza P.O. 12622, Egypt; 3 Physics Department, Faculty of Science, Cairo University, Giza P.O. 12613, Egypt; mfikry@sci.cu.edu.eg; 4 Chemistry of Natural and Microbial Products Department, Pharmaceutical and Drug Industries Division, National Research Centre, 33 El Bohouth St. (Former El Tahrir St.), Giza P.O. 12622, Egypt; yabdelrahman11@yahoo.com (Y.M.S.); sweed_ayman@yahoo.com (A.M.K.S.); 5 Medicinal Chemistry, Trinity Translational Medicine Institute, Trinity Centre for Health Sciences, Trinity College Dublin, The University of Dublin, St. James’s Hospital, Dublin 8, Ireland

**Keywords:** *m*THPP, ethylcellulose, chitosan, nanocomposite, multidrug resistance, photodynamic therapy, antimicrobial, laser light

## Abstract

The development of new antimicrobial strategies that act more efficiently than traditional antibiotics is becoming a necessity to combat multidrug-resistant pathogens. Here we report the efficacy of laser-light-irradiated 5,10,15,20-tetrakis(*m*-hydroxyphenyl)porphyrin (*m*THPP) loaded onto an ethylcellulose (EC)/chitosan (Chs) nanocomposite in eradicating multi-drug resistant *Pseudomonas aeruginosa*, *Staphylococcus aureus*, and *Candida albicans.* Surface loading of the ethylcelllose/chitosan composite with *m*THPP was carried out and the resulting nanocomposite was fully characterized. The results indicate that the prepared nanocomposite incorporates *m*THPP inside, and that the composite acquired an overall positive charge. The incorporation of *m*THPP into the nanocomposite enhanced the photo- and thermal stability. Different laser wavelengths (458; 476; 488; 515; 635 nm), powers (5–70 mW), and exposure times (15–45 min) were investigated in the antimicrobial photodynamic therapy (aPDT) experiments, with the best inhibition observed using 635 nm with the *m*THPP EC/Chs nanocomposite for *C. albicans* (59 ± 0.21%), *P. aeruginosa* (71.7 ± 1.72%), and *S. aureus* (74.2 ± 1.26%) with illumination of only 15 min. Utilization of higher doses (70 mW) for longer periods achieved more eradication of microbial growth.

## 1. Introduction

The rapidly growing resistance of microbial pathogens against antibiotics must be considered as one of the most significant clinical challenges facing the world nowadays [1,2]. The development of microbial resistance against most of the known classes of antibiotics has become an acute problem, particularly in hospitals [3,4]. Likewise, in the last few decades, advances in the search for new antibiotics have not kept pace with the growing number of resistant bacterial strains [1]. For example, many reports showed that extended-spectrum β-lactamase-producing Enterobacteriaceae (ESBL-PE), multidrug-resistant (MDR) *Pseudomonas aeruginosa*, carbapenem-resistant Enterobacteriaceae (CRE), *Acinetobacter baumannii*, and methicillin-resistant *Staphylococcus aureus* (MRSA) are increasingly identified as the predominant causative pathogens in patients, due to antibiotic misuse [1,5] Therefore, to counteract this emerging public health problem there is an urgent need to develop and identify new antimicrobial strategies that are non-incursive, non-toxic, and more effective than the current antibiotics [1,2,3].

Antimicrobial photodynamic therapy (aPDT) is one of the longest known and most advanced procedures to counteract different pathogens [1,6,7]. It relies on the standard PDT principle of applying a nontoxic dye, which upon absorption of light generates singlet oxygen and other reactive oxygen species via photosensitization. Illumination with laser light allows the excitation wavelength to be matched with the absorbance maxima of the photosensitizer. This process minimizes the overheating of the tissue with laser light. Moreover, the laser light can easily be focused into optical fibers and transported over long distances inside the body cavities or through the lumens of needles into illuminated tissue [8,9,10]. Additionally, certain laser wavelengths (from red light to NIR) are preferable for aPDT due to the thermal and penetration interactions with microbes, antimicrobial agents, and/or tissue [11,12].

Both Gram-positive and Gram-negative bacteria have been shown to be susceptible to photodynamic action under certain conditions in the presence of photosensitizers and irradiation [1,6,13,14]. Recently, aPDT has become an alternative technique for the eradication of multi-drug resistant microorganisms, and we are interested in compounds related to approved photosensitizers such as 5,10,15,20-tetrakis(*m*-hydroxyphenyl)chlorin (*m*THPC) [15]. For example, it has been reported that (5,10,15,20-tetrakis(*p-*hydroxyphenyl)porphyrin (*p*THPP) conjugated with the biopolymer chitosan (Chs) has photomicrobial activity against *Escherichia coli* in a water disinfection process [16]. Herein we explore for the first time—to our knowledge—the photomicrobial activity of (5,10,15,20-tetrakis (*m-*hydroxyphenyl)porphyrin (*m*THPP) loaded over ethylcellulose/chitosan nanocomposite against multi-drug resistant *Pseudomonas aeruginosa*, *Staphylococcus aureus*, and *Candida albicans*.

The compound *m*THPP shows low cytotoxicity in the dark, and strong photocytotoxicity. It is chemically pure, of known composition, and can be synthesized in good yield from inexpensive and commercially available starting materials. In addition, it has absorbance in the red region of the electromagnetic spectrum, where tissues are most transparent for light, and has a high singlet oxygen quantum yield [17]. However, it aggregates in aqueous media and is difficult to deliver into biological systems [18]. For this purpose, we have immobilized *m*THPP with natural polymeric supports in order to overcome the aggregation of *m*THPP and studied the aPDT efficacy of the designed nanocomposite using different laser wavelengths, powers, and exposure times [19,20].

Biopolymers are polymeric materials produced by living organisms [21], which include polysaccharides, and are characterized by their bioavailability [22], biodegradability, and sustainability [23]. In this regard, cellulose and its derivatives are excellent examples of biopolymers, as cellulose is the most abundant biopolymer on earth [24,25,26,27,28]. Ethylcellulose is one of the ether cellulose derivatives, which is non-toxic and has a good safety profile [29]. It is recommended by the US Food and Drug Administration (FDA) as a safe and acceptable non-medicinal ingredient for use in oral capsules, suspensions, tablets, topical emulsions, and vaginal or ocular preparations [30]. Chitosan is another example, which is antimicrobial, biocompatible, and has a high compatibility grade with human body fluids without any allergenic reaction [31]. It has been reported that loading the target photosensitizer over cellulose material through conjugation or even by surface absorption enhances the photobacterial surface and hence reduces the bacterial cell count [32]. It is also well-established that Gram-positive bacteria and especially Gram-negative bacteria are susceptible to cationic photosensitizers [33]. For these reasons, we combined the *m*THPP, cellulose, and chitosan as a cationic component [34] in a composite in the nanostructure to explore their photocytotoxicity against the three organisms under study. In addition, the drug delivery systems in the nanostructures based on polysaccharide materials enhance the features of the loaded drug, e.g., bioavailability and durability [35]. Additionally, the microstructure of polysaccharides usually impacts drug efficiency through disaggregation of drug particles into the 3D network [36,37,38].

The focus of the present study is the surface loading of EC/Chs nanocomposite with *m*THPP, yielding the *m*THPP EC/Chs nanocomposite. Characterizations of the resulting nanocomposite were performed with FT-IR, X-ray diffraction (XRD), and scanning electron microscopy (SEM). Screening of different continuous laser systems with different powers allowed the investigation of the effects of different laser wavelengths to select the most effective option for the development of using this nanocomposite for aPDT against multi-drug resistant microbial cells.

## 2. Results and Discussion

### 2.1. Preparation of mTHPP-Loaded Nanocomposite

**Figure 1:**
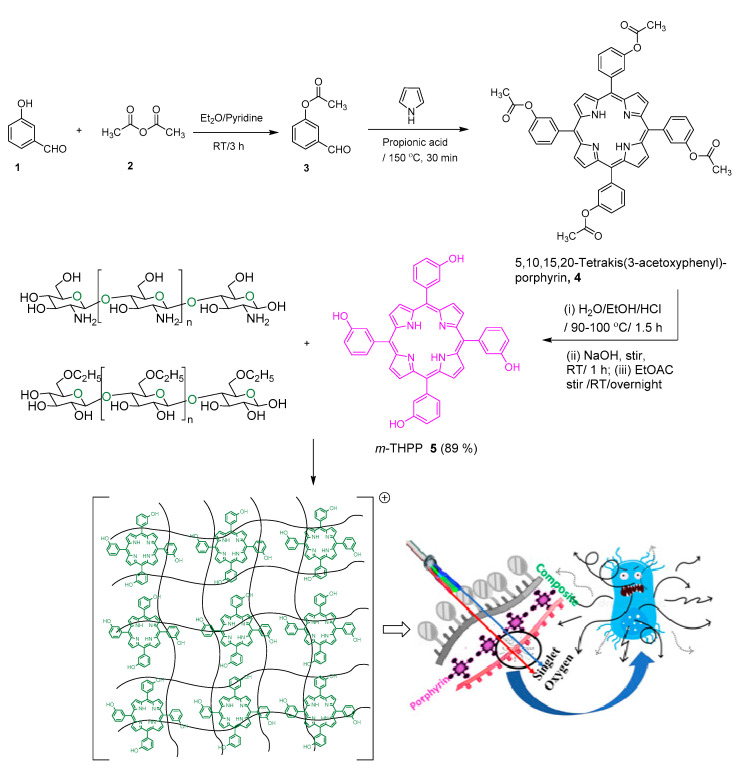
Design and synthesis of *m*THPP EC/Chs nanocomposite for aPDT.

The antimicrobial photodynamic therapy (aPDT) of *m*THPP-loaded EC/Chs nanocomposite was investigated for three pathogens. In the present study, the *m*THPP photosensitizer was chosen due to its absorption capacity at several laser light wavelengths in the blue and red region, which allows for the investigation of the effect of different excitation wavelengths. The synthesis of *m*THPP was achieved using the standard procedure (Figure 1) and was followed by loading it into EC/Chs nanocomposite to overcome its solubility drawbacks and enhance drug delivery [39]. The resultant *m*THPP EC/Chs nanocomposite possesses a green color, which indicates that the system acquired an overall positive charge. Chitosan was suggested to be the source of the positive charge, and this is compatible with the literature [39]. Zeta measurements in Table 1 proved an overall positive charge for the nanocomposite.

### 2.2. Characterization of mTHPP and mTHPP-Loaded Nanocomposite

#### 2.2.1. Polarized Light Microscopy

**Figure 2:**
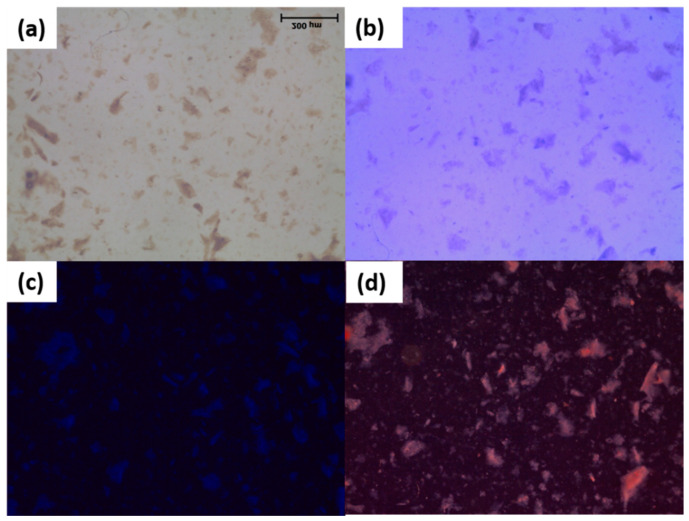
Polarized microscope image for the *m*THPP-loaded nanocomposite. (**a**) No filter; (**b**) 530 nm filter; (**c**) cold blue light; (**d**) daylight cold blue filter.

Polarized light microscopy was used to evaluate the light activity of the *m*THPP-loaded nanocomposite by applying different filters, as shown in Figure 2. The light-reflection pattern of *m*THPP was assigned in daylight with a cold blue filter, which indicated incorporation of *m*THPP into the nanocomposite structure.

#### 2.2.2. FT-IR Spectroscopy

**Figure 3:**
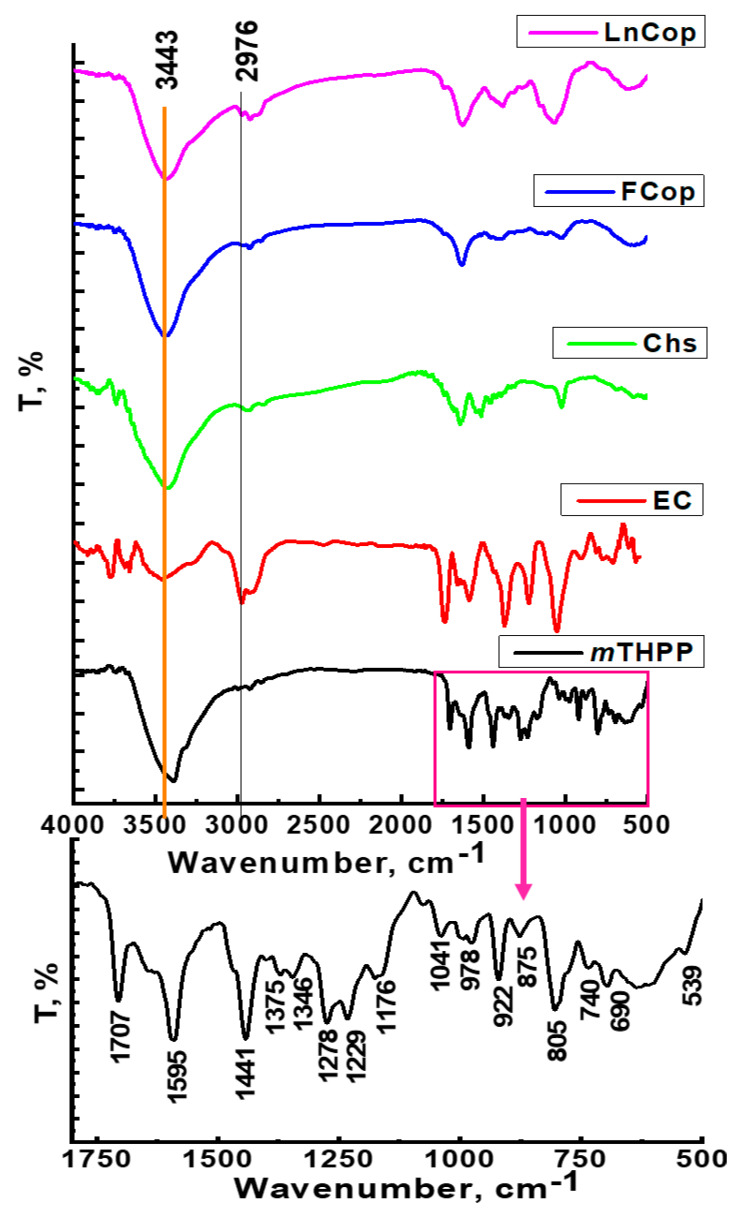
FT-IR of *m*THPP, EC, Chs, free composite, and *m*THPP-loaded nanocomposite.

The FT-IR spectra of the parent materials, free composite, and *m*THPP EC/Chs nanocomposite are shown in Figure 3. The *m*THPP spectrum displays a specific region of *m*THPP between 1700–500 cm^−1^. The characteristic bands of *m*THPP at 3386, 3021, 2928, 1596, 1441, 978, 740, and 559 cm^−1^ are associated with NH stretching, CH (phenyl) stretching, CH (pyrrole) stretching, the vibration of C–C–C in phenyl rings, CH rocking on phenyl, in-plane δ and out-of-plane δ N-H, and hydrogen atom motion (NH), respectively [40,41]. The EC spectrum revealed specific bands that are characterized for the cellulose derivatives. The bands at 3453, 2976, 2481, 1363, and 1051 cm^−1^ are associated with OH group stretching, H group stretching, H–C–H asymmetric stretching, symmetric stretching of terminal CH_3_ of a primary ethyl group, C–H bending, and C–O–C stretching, respectively [42,43]. Additionally, the FT-IR spectrum of chitosan revealed bands at 3471, 2948, 2840, 1644, 1514, and 1025 cm^−1^, indicative of N–H stretching, symmetric CH_3_ and asymmetric CH_2_ stretching, CH stretching, C=O stretching (amide I), –NH stretching (amide II), and the free amino group (–NH_2_) at the C_2_ position of glucosamine, respectively [44]. On the other hand, the free composite was assigned a major band at 3411 cm^−1^, which corresponds to overlapping hydroxyl groups of the composite. Additionally, the intensity of the band at 2935 cm^−1^ was decreased and the position shifted to lower frequency. In addition, a peak at 1640 cm^−1^ became sharper. Moreover, upon the loading of *m*THPP into the composite, a significant change was that the band of the hydroxyl groups was shifted to lower frequency, and a band at 1381 cm^−1^ was shifted to the lower frequency resulting from terminal CH_3_ groups. Moreover, a new band at 1086 cm^−1^ was observed.

#### 2.2.3. XRD Analysis

**Figure 4:**
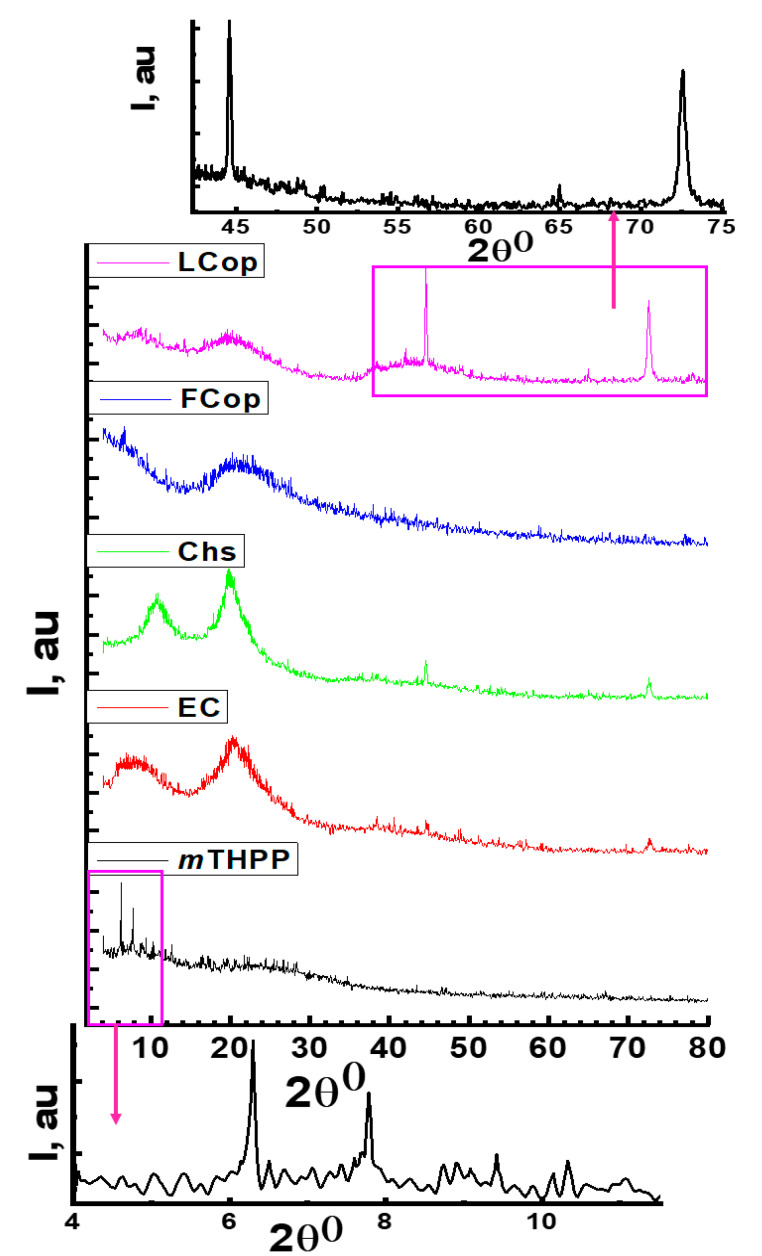
XRD pattern of *m*THPP, EC, Chs, free composite, and *m*THPP-loaded nanocomposite.

The XRD patterns of native materials, as well as free and loaded nanocomposites, are shown in Figure 4. The *m*THPP exhibits a typical diffraction pattern, as reported by Kang et al. [44]. Likewise, the polysaccharide derivatives exhibited a pattern characteristic for polysaccharides. Herein, the EC sample exhibited broad peaks at around 7.80° and a sharp band at 20.6°, which refers to a d-spacing at 10.8 and 4.33 Å respectively, like all cellulose and cellulosic derivatives [45]. In addition, the Chs sample showed a typical chitosan XRD pattern, which indicated two sharp peaks at 11 and 20° with d-spacing at 8.3 and 4.4 Å, respectively [46,47]. On the other hand, the peaks at 44 and 74° of the *m*THPP crystallographic isotropic phase were observed in the loaded nanocomposite as they had been observed in the magnified load nanocomposite pattern, which may be indicative of the inclusion of the *m*THPP particle into the composite network.

#### 2.2.4. Scanning Electron and Energy Dispersive Electron Spectroscopy

**Figure 5:**
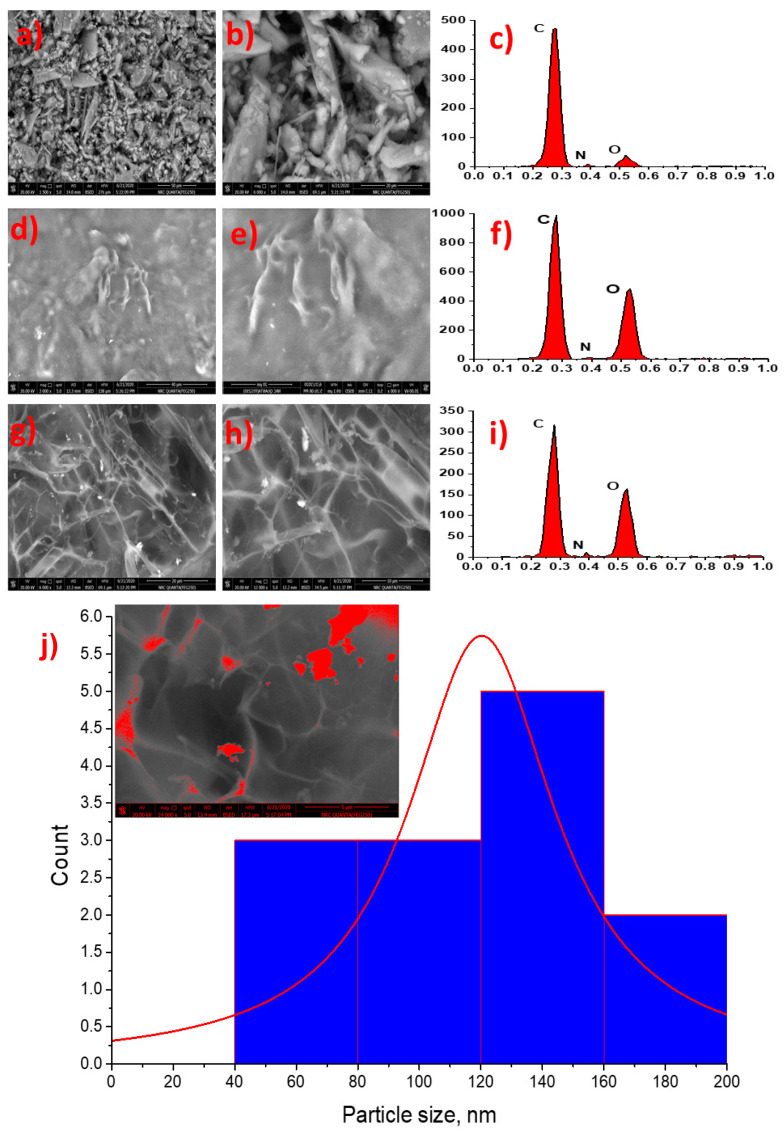
(**a**) Porphyrin at low magnification; (**b**) high magnification; (**c**) EDX.; (**d**) Free composite at low magnification; (**e**) high magnification; (**f**) EDX.; (**g**) Loaded nanocomposite at low magnification; (**h**) high magnification; (**i**) EDX.; (**j**) image process of the high magnification loaded nanocomposite SEM image.

Figure 5 illustrates the topographical studies of *m*THPP, the free composite, and the *m*THPP-loaded nanocomposite. The *m*THPP surface in Figure 5a,b shows a random surface appearance with EDX chart continent from C, N, and O. In contrast, the free composite appearance in low and high magnification in Figure 5d,e appears as a collapsed network with layer–layer constriction. On the other hand, the *m*THPP-loaded nanocomposite topography clearly shows a uniform network structure including *m*THPP particles in nanorange (>200 nm). Moreover, the imaging process of the high magnification SEM obtained to image the average particle size of *m*THPP and *m*THPP EC/Chs nanocomposite was recorded at about 120 nm. This confirms that the prepared carrier system is of nano scale.

#### 2.2.5. Particle Size and Zeta Potential Measurements

The dynamic light scattering (DLS) results, polydispersity index (PDI), and mean particle size determinations are listed in Table 1. The particle distribution was measured as a dispersion in aqueous solution and gave values of 175, 292, and 595 nm for the *m*THPP free composite, and *m*THPP-loaded nanocomposite, respectively. These particle sizes indicate that the free composite is of nanoscale dimension. However, the loading process resulted in more particle aggregation where the PDI values of *m*THPP EC/Chs nanocomposite was increased twofold. On the other hand, the zeta potential was calculated from the three main measured factors (cell current, phase shift, and mobility) that depend on the charge over particle surface. Zeta measurements of *m*THPP, free nanocomposite, and *m*THPP EC/Chs nanocomposite are tabulated in Table 1. Neat *m*THPP had a negative zeta charge, which presented an average zeta potential of –20.55 mV. For the free composite, a highly positive zeta charge was recorded and determined to be 35.5 mV. This is the result of chitosan, which plays a vital role in the net charge of nanocomposite; whereas EC was charge-neutral [48,49]. In addition, the *m*THPP-loaded nanocomposite system had a decreased positive charge of 23.84 mV.

#### 2.2.6. Thermal Stability and UV/Vis Absorption of *m*THPP and Its Nanocomposite

**Figure 6:**
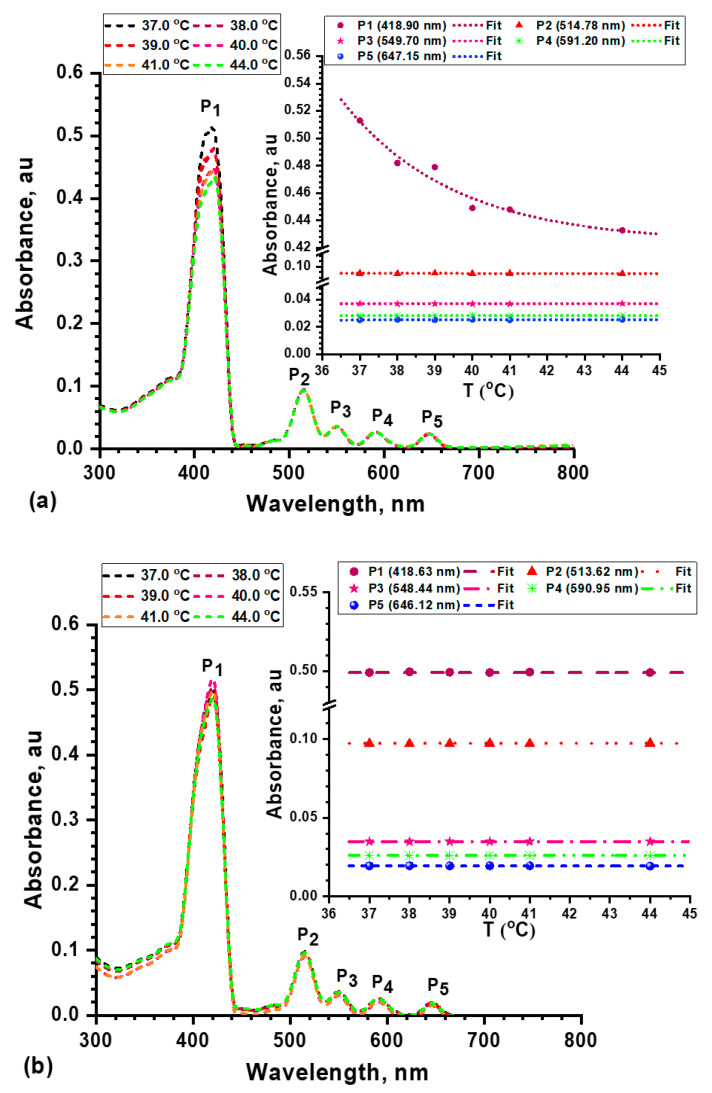
Absorption spectra (main curves) and the thermal stability of each peak (insert curves) of (**a**) porphyrin and (**b**) porphyrin nanocomposite at different temperatures.

The UV/Vis absorption of porphyrins and materials derived thereof is one of the main analytical tools in this area [50]. In addition, the thermal stability of the porphyrin and the nanocomposite were studied to assure their stability under biological conditions. The temperature of *m*THPP and *m*THPP-loaded nanocomposite solutions dissolved in DMSO was varied gradually from 37 to 44 °C. The absorption spectra of the porphyrin and its nanocomposite at different temperatures exhibit five absorption bands as shown in Figure 6a,b. The first one, P_1_ at 419 nm, represents the Soret or B band due to the transition from the ground state (S_0_) to the second excited state (S_2_), which ranges from 380 to 500 nm depending on whether the porphyrin is β- or *meso* substituted. The other bands are P_2_ at 515 nm, P_3_ at 550 nm, P_4_ at 591 nm, and P_5_ at 647 nm. These represent the Q bands, which range from 500 to 750 nm due to the weak transitions from S_0_ to the first excited state (S_1_) [51]. The relative absorbances for the porphyrin were 0.0064, 0.0112, 0.014, 0.0952, and 0.096; while the porphyrin nanocomposite had relative absorbances of 0.0078, 0.0133, 0.0155, 0.098, and 0.0082 for the laser wavelengths used—458, 476, 488, 515, and 637 nm, respectively. Accordingly, the descending arrangement of the laser wavelengths according to their absorption by the porphyrin and its nanocomposite is 515 nm > 488 nm > 476 nm > 635 nm > 458 nm. The porphyrin nanocomposite has different absorbance enhancement ratios for P1, P2, P3, and P4, such as 22%, 18%, 11%, and 3%, respectively for the laser wavelengths used, while the absorbance of P5 is quenched by 15%. The inset of Figure 6a,b shows that the porphyrin absorption bands are unaffected by the temperature except for P_1_ at 418.90 nm, which exhibits an absorbance decrease from 0.513 to 0.432 upon the slight temperature increase. The porphyrin nanocomposite enhanced the thermal stability of the porphyrin absorption P_1_.

#### 2.2.7. Photodynamic Inactivation of Microbial Strains

The current study aimed to determine the degree to which photodynamic therapy using *m*THPP-loaded nanocomposites could be effective against multidrug-resistant pathogens in vitro. The microwell dilution method based on turbidometry and the plate counting method (CFU method) were used to prescreen the antimicrobial efficacy of *m*THPP and the *m*THPP-loaded nanocomposite in the presence (irradiation with 635 nm laser light) and in the absence of laser light.

As shown in Table 2, both the *m*THPP and *m*THPP-loaded nanocomposite significantly suppressed the growth of bacterial (*P. aeruginosa,* and *S. aureus*) and yeast cells (*C. albicans*) when exposed to laser light, as compared to the absence of laser light. In the presence of *m*THPP and *m*THPP-loaded nanocomposite alone in the dark, the inhibiting efficiencies were less than 7% and 3%, respectively. These preliminary results showed significant photocytotoxicity efficacy of the EC/cellulose/mTHPP nanocomposite at (635 nm) and low cytotoxicity in the dark against three multi-resistant pathogens, which represented that the designed nanocomposite could be introduced as a good candidate photosensitizer for aPDT application.

Accordingly, in more in-depth studies, the laser light was applied only and in combination with the *m*THPP or *m*THPP-loaded nanocomposite against the microbial pathogens at 50 µg/mL (dissolved in dimethyl sulfoxide (DMSO) at different wavelengths (458, 476, 488, 515, 635 nm), at the power of 5 mW/cm^2^, and at different exposure times (15, 30, 45 min).

As can be seen in Table 3, *m*THPP and the *m*THPP-loaded nanocomposite provided a potential photodynamic effect when excited with laser light. In the case of irradiation alone (without *m*THPP or *m*THPP-loaded nanocomposite), it was evident that the microbial survival rate decreased close to the blue irradiation region (458–488 nm). E.g., the *P. aeruginosa*, and *S. aureus* survival rates were reduced with 32.8 ± 0.94% and 27.7 ± 1.16% at 476 nm and 488 nm (exposure for 30 min), respectively. Notable photoinactivation of *C. albicans* was observed as 23.6 ± 1.45% at 458 nm and irradiation for 30 min. Upon using longer wavelengths, the reduction of microbial survival was decreased to 7.33 ± 0.94%, 10.5 ± 0.62%, and 7.8 ± 0.32% at 635 nm (red-light region) for *C. albicans*, *P. aeruginosa*, and *S. aureus*, respectively. These results screened the efficacy of laser illumination only on the three organisms for 15–30 min at different wavelengths between 458–635 nm. The study showed that the blue light (458, 476, and 488 nm) significantly reduces the microbial survival rate of the three organisms. However, the red light showed threefold less effectiveness than the blue light, indicating that the red light is safe and does not affect the endogenous chromophores.

The irradiation group of *m*THPP with different laser light wavelengths for each microorganism indicated the combined effect of illumination and porphyrin. The degree of photoinactivation in the blue-light region was more pronounced than under red light illumination. Obviously, *P. aeruginosa* was strongly photoinactivated under blue light irradiation for 15 min with growth inhibition of 53.1 ± 2.3% and 65.8 ± 2.23% at 476 nm and 488 nm, respectively. The photoinactivation rate of *S. aureus* reached 54.06 ± 0.82% and 47.2 ± 0.55% at 476 nm and 488 nm, respectively. Indeed, the action of *m*THPP supplemented with laser light for 15 min significantly suppressed bacterial growth (65.8 ± 2.23% for *P. aeruginosa* at 488 nm, and 54.06 ± 0.82% for *S. aureus* at 476 nm) about three- and eightfold as compared to the irradiation group only (irradiated for 15 min). Furthermore, the maximum inhibition of *C. albicans* by the irradiated *m*THPP was achieved at 458 nm with 44.03 ± 0.75%, which showed a threefold efficiency as compared to the irradiation group only (13.3 ± 0.5%). Herein, we screened the efficacy of *m*THPP when illuminated at different wavelengths (458–635), which showed a potential reduction in bacterial growth for the blue-laser-light region and to a lesser extent for red laser light.

Irradiation of *m*THPP-loaded nanocomposite had a lesser effect than *m*THPP on bacterial growth under blue laser light irradiation at 476 nm for 15 min (37.9 ± 2.07% for *P. aeruginosa*, and 30.8 ± 1.04% for *S. aureus*), and at 488 nm for 15 min (41.7 ± 1.02% for *P. aeruginosa*, and 42.2 ± 2.17% for *S. aureus*). While a lower survival reduction of *C. albicans* with 21.8 ± 1.02%, 21.9 ± 0.89%, and 17.5 ± 1.23% was emphasized upon exposure at 458 nm, 476 nm, and 488 nm for 15 min. Notably, there was lower effect of irradiation of *m*THPP-loaded nanocomposite on *C. albicans* survival, which resulted in 27.2 ± 1.39 growth reduction as compared to 23.6 ± 1.45% for the irradiated group alone. Surprisingly, irradiation of the loaded nanocomposite in the red region (635 nm for 15 min) more efficiently suppressed the microbial survival than in the blue-light region (458–488 nm) with 71.7 ± 1.72%, 74.2 ± 1.26%, and 59.1 ± 0.21% for *P. aeruginosa*, *S. aureus,* and *C. albicans*, respectively. However, irradiation of *m*THPP under red laser light gave less photoinactivation than that achieved by the nanocomposite at the same exposure time (15 min), whereas the reduction of *P. aeruginosa*, *S. aureus*, and *C. albicans* survival at 635 nm was not more than 36.06 ± 0.82%, 52.3 ± 0.49%, and 35.06 ± 2.2%, respectively. In general, the photoinactivation of irradiated *m*THPP was found to be more pronounced on the microbial survival inhibition than irradiated *m*THPP-loaded nanocomposite in the blue-light region. Conversely, the irradiation of the *m*THPP-loaded nanocomposite under the red-light region was more efficient than the irradiated *m*THPP under the same light region. In other words, introduction of the nanocomposite dramatically reduced the bacterial survival rate in the therapeutic window of the electromagnetic spectrum (600–800) in only 15 min (Table 3).

Our findings show that both *m*THPP and the *m*THPP-loaded nanocomposite exert significant antimicrobial susceptibility only with irradiation by laser light; and the microbial eradication efficiencies of the composite are low in the absence of light. The synergistic effect of photodynamic inactivation and antimicrobial activity of the composite material was most effective upon irradiation with blue light. The UV/Vis absorption (Figure 6) showed no differences between the absorption bands of *m*THPP and *m*THPP-loaded nanocomposite, but clear differences were found in the photoinactivation performance of *m*THPP and *m*THPP-loaded nanocomposite between irradiation with blue (458–486 nm) and red (635 nm) light. This variation may be attributed to the fact that the red light had more penetration into ethylcellulose/chitosan (mimicking biological tissue) than the blue light [6,52]. The efficient distribution of *m*THPP in the nanocomposite network (Figure 1) increases the surface area of photoactive pigments, hence making the system more active.

There are several explanations why blue light irradiation is more effective than red light on microbial cells. Blue light may activate endogenous photosensitizers in the microbial cells resulting in the generation of reactive oxygen species (ROS) [5]. Secondly, irradiation with blue light may increase the free radical release [5,14]. Following our findings, irradiation of *Escherichia coli* at 415 nm in the absence of photosensitizers resulted in a 30% reduction in survival [53]. Similarly, exposure of *S. aureus* (Gram-positive), *E. coli* (Gram-negative), and *C. albicans* (yeast-like fungi) to laser irradiation in blue region at 405 and 445 nm for 45 min inhibited their growth by more than 50% without supplementation of exogenous photosensitizer [54].

Even though blue light irradiation reduces microbial survival without an exogenous photosensitizer, the inclusion of an exogenous photosensitizer in our design offers several advantages. Without an exogenous photosensitizer, long irradiation times are required (45 min) to reach at least 50% inhibition. Light penetration of blue light is lower than that of red light, and photodynamic therapy with red light diminishes reactions with human cells and the activation of endogenous photosensitizers.

Next, we optimized the laser power for each preferred wavelength to enhance the microbial photoinactivation of the *m*THPP-loaded nanocomposite. In this regard, a suitable wavelength for each microorganism was used with different powers (10, 20, 40, 70 mW/cm^2^) at a fixed concentration of *m*THPP and the *m*THPP-loaded nanocomposite (50 µg/mL). As shown in Table 4, the irradiation power makes a significant difference in the reduction rate of microbial survival in the blue-light region, particularly in the case of *C. albicans*. The photoinactivation efficiency for *C. albicans* at 458 nm for 15 min was increased upon an increase in irradiation power and reached up to maximum values 83.66 ± 1.24 and 56.13 ± 0.69% at 70 mW/cm^2^ for *m*THPP and *m*THPP-loaded nanocomposite, respectively. In addition, the photoinactivation of *P. aeruginosa* was notably enhanced upon a raise in irradiation power (83.4 ± 0.53% for *m*THPP and 59.2 ± 0.90% for *m*THPP-loaded nanocomposite at 70 mW/cm^2^). Similar results were obtained for the photoinactivation of *S. aureus*.

Note that extending the exposure time of the appropriate light wavelength was found to be more efficient for microbial cell eradication (Table 4). The count of viable microbial colonies was significantly decreased with longer times, with bacterial survival irradiated in the presence of *m*THPP for longer exposure time (45 min) reaching 92% and 94.7% for *P. aeruginosa* (at 476 nm and 70 mW/cm^2^) and *S. aureus* (at 488 nm and 70 mW/cm^2^), respectively. Likewise, with longer exposure times the *m*THPP-loaded nanocomposite somewhat increased photoinactivation, especially under red light. Apparently, the effect of higher energy power on *C. albicans* suggests that higher doses may be necessary to promote further absorption, which is known to be faster in prokaryotic than in eukaryotic cells [54]. Overall, the Gram-positive bacterium was more sensitive to photosensitizers at lower doses than Gram-negative bacteria.

Many photosensitizers effective for the eradication of microbes carry or are conjugated with functional groups that give it a positive charge. For example, it has been reported that cationic porphyrins provide effective photoinactivation of both Gram-positive and Gram-negative bacteria by promoting photosensitizer attachment to the microbial cell wall and ROS production [4,6,14]. Likewise, many studies reported more effective photoinactivation by forming porphyrin-conjugates. E.g., a porphyrin conjugate with mannose–hPG provided eradication of *S. aureus* under red light (655 nm) [55]. Similarly, it has been reported that the cationic porphyrin–polymyxin B conjugate has higher bacterial absorption than neutral porphyrins [56]. In agreement, conjugation of *m*THPP and ethylcellulose/chitosan and its overall cationic nature gave significant antimicrobial activity under red light irradiation.

#### 2.2.8. Minimal Inhibitory Concentration and Minimal Bactericidal Concentration

To determine the minimal inhibition concentration (MIC) and minimal bactericidal inhibition (MBC), various concentrations (25–400 µg/ mL) of *m*THPP and *m*THPP-loaded nanocomposite were examined under an appropriate wavelength and power for each microorganism using exposure times of 30 min. Irradiation of *m*THPP under blue light gave of MIC at 50 and 25 µg/mL, and MBC of 100 and 200 µg/mL for *P. aeruginosa* and *S. aureus*, respectively. On the other hand, the *m*THPP-loaded nanocomposite gave MIC of 50, and 100 µg/mL, and MBC of 100 and 200 µg/mL for *P. aeruginosa* and *S. aureus*, respectively. In addition, the MIC of *C. albicans* was determined to be 100 and 50 μg/mL for irradiated *m*THPP under blue light and *m*THPP-loaded nanocomposite under red light, while the MBC value of the irradiated *m*THPP and *m*THPP-loaded nanocomposite was found to be 200 μg/mL.

## 3. Materials and Methods

### 3.1. Materials

Microbial pathogens were kindly donated by the Microbiology and Immunology Department, Faculty of Medicine (boys), Al-Azhar University (Cairo, Egypt) (Ethylcellulose and chitosan used in this study were purchased from Sigma Aldrich (St. Louis, MO, USA), molecular weight 650,000, viscosity, 275.9 cps, and degree of deacetylation, 85.5%). Nutrient agar and potato dextrose agar media were purchased from Conda Lab (Madrid, Spain).

### 3.2. Preparation of Photosensitizer Nanocomposite

#### 3.2.1. Synthesis of *m*THPP

5,10,15,20-Tetrakis(3-hydroxyphenyl)porphyrin, *m*THPP was prepared by the Adler condensation method [39] in which pyrrole, 3-acetoxy benzaldehyde were allowed to reflux in propionic acid for 30 min followed by hydrolysis of the ester group according to a literature procedure [57,58].

#### 3.2.2. Composite Preparation

Chitosan (0.2 g) was dissolved in 50 mL (1% acetic acid solution). Ethylcellulose (0.3 g) was dissolved in 50 mL ethanol. The two solutions were miscible under vigorous stirring at room temperature for one hour. The temperature was raised to 70 °C and the mixture was stirred at 1500 rpm overnight. The prepared composite was ultrasonicated for 5 min and stored in a refrigerator (10 °C) for further observations.

#### 3.2.3. Loading of *m*THPP onto the Nanocomposite

*m*THPP (0.05 g, 0.074 mmol) was dissolved in 5 mL ethanol. The *m*THPP solution was added dropwise to the previously prepared composite (100 mL) under stirring at 1500 rpm overnight.

### 3.3. Characterizations and Instrumentation

*m*THPP and *m*THPP-loaded nanocomposites were characterized via several techniques using the following instruments: polarized microscope, Leica Microsystems (Switzerland) Ltd. (NRC, Giza, Egypt) (Leica DM750P); FT-IR spectrometer (NRC, Giza, Egypt) (Nicolet Impact-400 FT-IR spectrophotometer) in the range of 400–4000 cm^−1^. A topographical study was carried out using scanning electron microscopy (SEM) with energy dispersive electron spectroscopy (EDX) (JSM 6360 L V, JEOL/Noran, Tokyo, Japan). For surface morphology imaging, different samples were recorded using an accelerating voltage of 10–15 kV. Additionally, the obtained images were processed via image J free software. The X-ray diffraction (XRD) (NRC, Giza, Egypt) of samples was investigated on a Diano X-ray diffractometer using a CuKα radiation source energized at 45 kV and a Philips X-ray diffractometer (PW 1930 generator, PW 1820 goniometer) with CuKα radiation source (λ = 0.15418 nm). The XRD patterns were recorded in a diffraction angle range of 2θ from 10° to 80° in reflection mode. The particle size distribution and zeta potential of the composite, *m*THPP, and *m*THPP-loaded nanocomposite were measured using Nicomp TM 380 ZLS size analyzer, Entegris, Billerica, MA, USA. Laser light scattering was used at 170° in the case of particle size detection, whereas zeta potential was measured at 18°.

UV/Vis absorption spectra of the porphyrin and composite solutions samples were measured using an Ocean Optics spectrometer (HR4000 UV–NIR) (Cairo University, Giza, Egypt) with a spectral resolution of 1 nm and equipped with an optical fiber (SMA 905). The absorption spectra ranged from 200 to 1100 nm. The liquid sample was set in a quartz cuvette in a dark chamber thermo-plate holder. The thermo-plate temperature was varied from 37 to 44 ℃ using a circulating water bath controller (MGW, Lauda M3, USA).

Laser irradiation experiments were carried out using two different continuous laser systems to investigate the effects of the laser wavelengths. A continuous argon laser system (Spectra-Physics, Model:183-C0201) with multi-wavelengths (458, 476, 488, and 515 nm) and multiple powers (from 10 to 70 mW), and a continuous diode laser (LAP Laser Applikationen, Model: LAP 5MDL-63) with a wavelength of 635 nm and power 5 mW were used.

### 3.4. Microorganisms and Culture Conditions

The microorganisms used in the study were methicillin-resistant *Staphylococcus aureus* (MRSA), multidrug resistant *Pseudomonas aeruginosa* (MDR-PA), and *Candida albicans* ATCC 10231. Before each experiment, the strains were cultivated aerobically in 20 mL of nutrient broth (*S. aureus*, *P. aeruginosa*) at 37 °C and potato dextrose broth (*C. albicans*) at 28 °C for 24 h while shaken. All the experimental procedures were performed under aseptic conditions [59,60]. *S. aureus* MRSA was susceptible to vancomycin but completely resistant to the other antimicrobial drugs. *Pseudomonas aeruginosa* (MDR-PA) was moderately susceptible to cefepime and highly resistant to other antibiotics tested.

### 3.5. Photodynamic Inactivation of Microbial Strains

The suspensions of bacteria (10^6^ CFU/mL) and yeast (10^8^ CFU/mL) were tested in 96-well microplates (GAMA GROUP, Czech Republic) by transferring each sample to Muller Hinton broth consisting of (g/L), beef extract (2); acid hydrolysate of casein (17.5); starch (1.5). The samples were divided into four test groups: (1) light only: microbial samples were exposed to various wavelengths of light in the absence of the PSs and composite. (2) The *m*THPP or *m*THPP-loaded nanocomposite only: microbial samples were treated in the presence of *m*THPP or *m*THPP-loaded nanocomposite and not irradiated. (3) Both *m*THPP or *m*THPP-loaded nanocomposite and light: microbial samples had *m*THPP or *m*THPP-loaded nanocomposite as in group 2 but were subsequently treated using light of an appropriate wavelength as in group 1 for different times (15–45 min). (4) Controls using microbial samples not treated by either laser light, or *m*THPP or *m*THPP-loaded nanocomposite.

The *m*THPP and *m*THPP-loaded nanocomposite were first added to the Muller Hinton medium with constant concentration (50 µg/mL). The microplates were initially irradiated with different wavelengths of light as described above for 15–45 min. The efficacy of *m*THPP and *m*THPP-loaded nanocomposite to induce the photoinactivation mechanism for the tested pathogens was evaluated by the turbidometry method and by the CFU plate method [53]. The experiments were repeated at least three times. The control samples were both microbial cells growing in the absence of each treatment and those exposed to light only (groups 1 and 4).

### 3.6. Determination of Microbial Survival

The survival of microbial cells following illumination was determined by counting their viable number (i.e., the CFU plate method) after 15 to 45 min exposure of the suspended bacteria and yeast to light. Microbial cultures grown under the same conditions but without light exposure served as controls. The absorbance of the suspension at 630 nm was monitored at predetermined periods to determine growth curves. The percent of growth inhibition and cell survival in the treated and untreated cells that were monitored via the turbidometry method was calculated using the following equation:

A − B/A × 100(1)

where A and B are the absorbances of the untreated and treated samples, respectively.

On the other hand, microbial survival in CFU/mL was calculated according to the following formula:

**CFU/mL** = Number of colonies in plate × Dilution factor/Amount transferred to plate (mL) (2)

The decrease in survival in all groups was calculated as a percentage, as shown below:

**Percent reduction** = [(Number of bacteria in control group CFU/mL) − (Number of bacteria in application group CFU/mL)] × 100/(Number of bacteria in control group CFU/mL) (3)

All experiments were repeated at least three times.

### 3.7. Determination of Minimal Inhibitory Concentration (MIC) and Minimal Bactericidal Concentration (MBC)

The MIC and MBC were determined by the microdilution method in 96-well microplates with modifications. Several concentrations of *m*THPP and *m*THPP-loaded nanocomposite (0.025–0.400 mg) were added in wells with Mueller Hinton broth (MHB) and the suspension with microorganisms. The positive control was a well with the bacterial suspension and MHB, while the negative control contained only MHB. A control with DMSO was also performed to discard the diluent activity. Three plates were produced in the same way; one plate was exposed for 20 min to red light irradiation and another plate was incubated under dark conditions (without any light exposure). Afterward, the plates were incubated for 24 h at 37 °C. The assay was revealed with CFU/mL of the microbial growth. The lowest concentration that did not show colony formation in the plate after treatment was considered as MIC. To determine the MBC, an aliquot of 10 μL was taken of each well, seeded on nutrient agar or a PDA plate, and incubated for 24 h. After the colonies were identified, the lowest concentration that did not demonstrate microbial growth was considered as the MBC [61].

Cytotoxicity of *m*THPP and *m*THPP-loaded nanocomposite was determined in vitro using Vero tissue culture: the MTT protocol [62,63] with minor modification. The Vero (normal fibroblast cell line) was obtained from the American-type culture collection (ATCC). The cell quantity and the percentage of the viable cell were calculated by the following formula:

**Viability%** = (Test OD)/(Control OD) × 100 (4)

**Inhibition%** = 100 − Viability% (5)

### 3.8. Cytotoxicity

**Figure 7:**
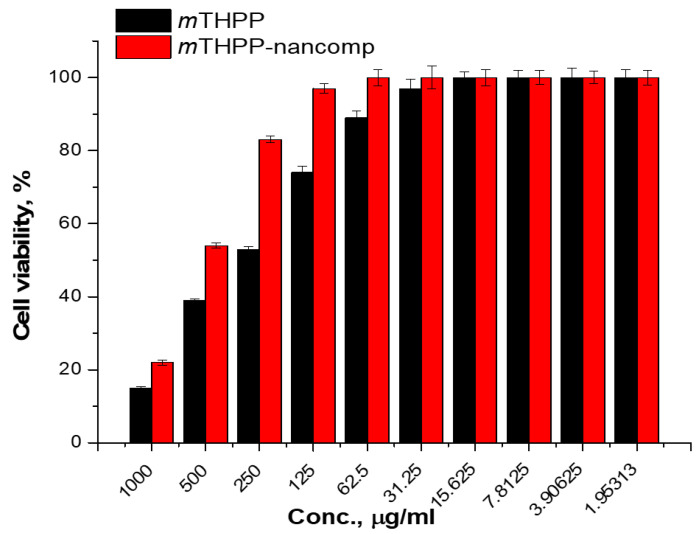
Cytotoxicity of *m*THPP and *m*THPP-loaded nanocomposite against a Vero normal cell line.

The cytotoxicity of *m*THPP and the *m*THPP-loaded nanocomposite against normal cell lines in vitro was considered as the first step to detect the safety of these products as presented in Figure 7. It is well-known that the *m*THPP has low cytotoxicity as reported in the literature [64]. Otherwise, the addition of biopolymer to the drug carrier system could decrease the cytotoxicity dose as predicted. Herein, the obtained results affirmed that the *m*THPP has a low cytotoxicity effect with IC_50_ of about 315 µg/mL and this was reduced to 475 µg/mL after loading into nanocomposite drug carrier system.

## 4. Conclusions

In this article, we designed and prepared *m*THPP EC/Chs nanocomposite for laser-induced aPDT against multidrug resistant pathogens *C. albicans*, *P. aeruginosa*, and *S. aureus*. The surface conjugation of the ethylcellulose/chitosan nanocomposite with the *m*THPP was proven and characterized by FT-IR, SEM, EDX, XRD, DLS, and UV–NIR absorption. The study of free *m*THPP when irradiated with laser light in the blue region (458, 488, and 476 nm) at a power of 70 mW for only 15 min revealed a significant reduction in the microbial survival rates of *C. albicans*, *P. aeruginosa*, and *S. aureus* (83.66 ± 1.24%, 83.4 ± 0.53%, 87.06 ± 0.87%, respectively). However, the *m*THPP EC/Chs nanocomposite reduced the microbial survival rates to a lesser extent (56.13 ± 0.69%, 59.2 ± 0.90%, 55.56 ± 0.80%, respectively) compared to *m*THPP under the same conditions. Conversely, introduction of ethylcellulose and chitosan biopolymer not only retained the activity of *m*THPP but also enhanced its physicochemical properties in the therapeutic red region (635 nm) of the electromagnetic spectrum. The new material *m*THPP EC/Chs nanocomposite showed a significant reduction in the microbial survival rates of *C. albicans*, *P. aeruginosa*, and *S. aureus* (59.1 ± 0.21%, 71.7 ± 1.72%, 74.2 ± 1.26%, respectively) upon illumination with 635 nm laser light for 15 min at 5 mW only. We conclude that the new material *m*THPP EC/Chs nanocomposite enhances the physicochemical and photo-killing properties of *m*THPP by utilizing the thermal and penetration properties of the therapeutic region of the electromagnetic spectrum. Note that increasing the power dose from 5 mW to higher clinical doses is expected to enhance the photokilling of the *m*THPP EC/Chs nanocomposite. The development of new porphyrin-based photosensitizer formulations for aPDT *m*THPP nanocomposites is a potential route for use in biological systems. Next, we will study the variation of clinical laser power, illumination time, and dose in the near-infrared region and will extend the biological samples to include other hospital-borne multi-drug resistant pathogens.

## Figures and Tables

**Table 1:** Zeta potential measurements of *m*THPP, the *m*THPP-loaded nanocomposite, and the free nanocomposite.

	Zeta Potential Measurements			Particle Size Measurements		
Sample	Cell Current, mA	Av. Phase Shift, rad/sec	Av. Mobility, M.U.	Av. Zeta Potential, mV	PDI	Average Particle Size, nm
*m*THPP	0.65	11.82	1.44	−20.55	0.137	175
Free composite	7.34	11.02	1.67	35.5	0.245	292
*m*THPP-loaded nanocomposite	1.65	18.2	2.48	23.84	0.442	595

**Table 2:** Screening of the antimicrobial activity of *m*THPP and *m*THPP-loaded nanocomposite in the presence and in the absence of red laser light (635 nm).

Sample	Inhibition in Microbial Survival, %		
	*P. aeruginosa*	*S. aureus*	*C. albicans*
*m*THPP	3.29 ± 0.19	2.15 ± 0.20	6.28 ± 0.54
*m*THPP-loaded nanocomposite	0.41 ± 0.02	0 ± 0	2.86 ± 0.11
*m*THPP (light)	21.76 ± 1.7	14.16 ± 0.91	10.6 ± 0.56
*m*THPP-loaded nanocomposite(light)	22.63 ± 1.5	16.5 ± 0.66	13.4 ± 0.40
Control	100		

**Table 3:** Antimicrobial photodynamic therapy of *m*THPP and *m*THPP-loaded nanocomposite at different wavelengths.

	Inhibition in *C. albicans* Survival %					
Wavelength	Laser Only		*m*THPP		*m*THPP-loaded Nanocomposite	
(nm)	Time Exposure (min)					
	**15**	**30**	**15**	**30**	**15**	**30**
458	13.3 ± 0.5	23.66 ± 1.45	44.03 ± 0.75	58.73 ± 1.11	21.8 ± 1.02	27.26 ± 1.39
476	10.36 ± 0.49	15.73 ± 0.87	36.03 ± 1.54	44.13 ± 0.71	21.93 ± 0.89	25.96 ± 0.77
488	13.93 ± 1.05	18.56 ± 1.14	38.33 ± 0.41	51.86 ± 0.49	17.53 ± 1.23	33.16 ± 0.26
515	7.33 ± 0.59	13.26 ± 1.47	40.1 ± 0.86	50.46 ± 0.40	31.26 ± 1.01	43.33 ± 0.74
635	3.46 ± 0.54	7.33 ± 0.94	35.06 ± 0.82	54.3 ± 0.64	59.1 ± 0.21	71.13 ± 1.5
Control	100					
	**Inhibition in *S. aureus* Survival %**					
**Wavelength**	**Laser Only**		***m*THPP**		***m*THPP-loaded Nanocomposite**	
**(nm)**	**Time Exposure (min)**					
	**15**	**30**	**15**	**30**	**15**	**30**
458	16.4 ± 0.60	21.7 ± 0.94	40.6 ± 1.22	74.4 ± 1.73	23.6 ± 1.47	32.6 ± 1.46
476	17.8 ± 0.32	22.7 ± 0.95	54.06 ± 0.82	83.4 ± 1.44	30.8 ± 1.04	38.3 ± 2.02
488	22.1 ± 1.3	27.7 ± 1.16	47.2 ± 0.55	70.6 ± 0.94	42.2 ± 2.17	56.3 ± 0.65
515	4.4 ± 0.45	10.3 ± 0.49	49.9 ± 0.91	56.7 ± 0.94	61.6 ± 0.75	72.8 ± 1
635	2.06 ± 0.12	7.8 ± 0.32	52.3 ± 0.49	58.1 ± 3.1	74.2 ± 1.26	81 ± 2.23
Control	100					
	**Inhibition in *P. aeruginosa* Survival %**					
**Wavelength**	**Laser Only**		***m*THPP**		***m*THPP-loaded Nanocomposite**	
**(nm)**	**Time Exposure (min)**					
	**15**	**30**	**15**	**30**	**15**	**30**
458	12.9 ± 1.06	34.2 ± 2.99	51 ± 2.94	79.3 ± 1.6	33 ± 1.51	36.5 ± 1.89
476	12.1 ± 0.84	32.8 ± 0.94	53.1 ± 2.31	75.9 ± 1.85	37.9 ± 2.07	43.8 ± 0.94
488	8.2 ± 0.28	27.3 ± 1.6	65.8 ± 2.23	82.2 ± 2.44	41.7 ± 1.02	53.7 ± 1.40
515	6.5 ± 0.74	20.3 ± 1.47	52.6 ± 1.94	63.8 ± 2.25	54.6 ± 1.32	63.5 ± 2.2
635	5.5 ± 0.97	10.5 ± 0.62	36.6 ± 2.27	59 ± 2.86	71.7 ± 1.72	83.1 ± 2.82
Control	100					

**Table 4:** Antimicrobial photodynamic therapy of *m*THPP and the *m*THPP-loaded nanocomposite at certain wavelengths with different irradiation powers.

Power (mW/cm^2^ at 458 nm)	Inhibition in *C. albicans* Survival %					
	Laser Only		*m*THPP		*m*THPP-loaded Nanocomposite	
	Time Exposure (min)					
	**15**	**30**	**15**	**30**	**15**	**30**
10	12.63 ± 0.70	23.36 ± 0.49	41.36 ± 1.14	60.96 ± 0.78	21.8 ± 0.86	27.6 ± 1.07
20	19.73 ± 0.65	29.2 ± 0.57	61.3 ± 0.49	72.3 ± 1.02	27.93 ± 0.65	32.33 ± 0.47
40	22.46 ± 1.1	33.26 ± 0.97	75.96 ± 0.85	77.33 ± 1.69	37 ± 1.10	41.33 ± 0.49
70	34.4 ± 0.43	47.96 ± 0.78	83.66 ± 1.24	85.2 ± 1.06	56.13 ± 0.69	60.43 ± 0.40
Control	100					
**Power** **(mW/cm^2^ at 476 nm)**	**Inhibition in *S. aureus* Survival %**					
	**Laser Only**		***m*THPP**		***m*THPP-loaded Nanocomposite**	
	**Time Exposure (min)**					
	**15**	**30**	**15**	**30**	**15**	**30**
10	18.13 ± 0.69	23.5 ± 0.57	34.71 ± 0.95	84.16 ± 0.70	23.46 ± 1.30	32.73 ± 0.74
20	21.06 ± 0.16	30.43 ± 0.75	56.3 ± 0.91	77.76 ± 1.5	35.96 ± 1.36	43.36 ± 1.08
40	23.9 ± 0.90	31.53 ± 1.12	76 ± 0.61	81.96 ± 1.36	41.26 ± 0.61	58.4 ± 0.43
70	30.5 ± 0.57	35.16 ± 0.30	87.06 ± 0.87	88.8 ± 0.69	55.56 ± 0.80	56.86 ± 1.29
Control	100					
**Power** **(mW/cm^2^ at 488 nm)**	**Inhibition in *P. aeruginosa* survival %**					
	**Laser Only**		***m*THPP**		***m*THPP-loaded Nanocomposite**	
	**Time Exposure (min)**					
	**15**	**30**	**15**	**30**	**15**	**30**
10	7.9 ± 0.24	26.96 ± 1	66.5 ± 0.5	82.96 ± 1.45	31.96 ± 0.85	36.8 ± 0.88
20	23.3 ± 0.57	36.83 ± 0.93	69.9 ± 0.82	88.13 ± 0.89	42.8 ± 0.88	44.43 ± 1.26
40	23.23 ± 1.01	37.73 ± 1.06	75.9 ± 0.77	87.86 ± 0.93	50.86 ± 0.69	59.4 ± 0.58
70	31.86 ± 0.93	44.03 ± 1.29	83.4 ± 0.53	90.26 ± 0.57	59.2 ± 0.90	62.26 ± 0.83
Control	100					

## Data Availability

The data presented in this study are available on request from the corresponding author.

## Sample Availability

Samples of the compounds are available from the authors.
